# The value of knowing: preferences for genetic testing to diagnose rare muscle diseases

**DOI:** 10.1186/s13023-024-03160-7

**Published:** 2024-04-22

**Authors:** Carol Mansfield, Marco Boeri, Josh Coulter, Eileen Baranowski, Susan Sparks, Kristina An Haack, Alaa Hamed

**Affiliations:** 1 Health Preference Assessment, RTI Health Solutions, Research Triangle Park, NC USA; 2 Health Economics and Value Assessment, Sanofi, Cambridge, MA USA; 3 Medical Affairs, Sanofi, Cambridge, MA USA; 4 Clinical development, Sanofi, Chilly-Mazarin, France

**Keywords:** Neuromuscular disease, Pompe disease, Genetic test, Diagnosis, Screening, Discrete-choice experiment

## Abstract

**Background:**

Genetic testing can offer early diagnosis and subsequent treatment of rare neuromuscular diseases. Options for these tests could be improved by understanding the preferences of patients for the features of different genetic tests, especially features that increase information available to patients.

**Methods:**

We developed an online discrete-choice experiment using key attributes of currently available tests for Pompe disease with six test attributes: number of rare muscle diseases tested for with corresponding probability of diagnosis, treatment availability, time from testing to results, inclusion of secondary findings, necessity of a muscle biopsy, and average time until final diagnosis if the first test is negative. Respondents were presented a choice between two tests with different costs, with respondents randomly assigned to one of two costs. Data were analyzed using random-parameters logit.

**Results:**

A total of 600 online respondents, aged 18 to 50 years, were recruited from the U.S. general population and included in the final analysis. Tests that targeted more diseases, required less time from testing to results, included information about unrelated health risks, and were linked to shorter time to the final diagnosis were preferred and associated with diseases with available treatment. Men placed relatively more importance than women on tests for diseases with available treatments. Most of the respondents would be more willing to get a genetic test that might return unrelated health information, with women exhibiting a statistically significant preference. While respondents were sensitive to cost, 30% of the sample assigned to the highest cost was willing to pay $500 for a test that could offer a diagnosis almost 2 years earlier.

**Conclusion:**

The results highlight the value people place on the information genetic tests can provide about their health, including faster diagnosis of rare, unexplained muscle weakness, but also the value of tests for multiple diseases, diseases without treatments, and incidental findings. An earlier time to diagnosis can provide faster access to treatment and an end to the diagnostic journey, which patients highly prefer.

**Supplementary Information:**

The online version contains supplementary material available at 10.1186/s13023-024-03160-7.

## Background

The variability of presenting symptoms and rarity of the disorders present a challenge for diagnosing neuromuscular diseases [1]. The inherent challenges associated with differential diagnoses and seeing multiple clinicians can cause delays in diagnoses and patient care [2]. These delays can lengthen a patient’s diagnostic journey to years in duration, with a corresponding burden on the patient and their family [2]. The diagnostic burden on patients can be alleviated by genetic testing, which reduces the time from diagnosis to treatment, minimizes invasive diagnostic testing (e.g., cerebrospinal fluid testing, electrodiagnostic testing, muscle/nerve biopsy), and improves the accuracy of information on genetic risks that allows informed family planning decisions [3, 4]. However, testing for conditions without available treatment may cause distress to patients and therefore should be approached with caution. Similarly, genetic tests may return results about unrelated conditions or information that is unclear and not actionable. Determining the amount of reimbursement for panel tests and how patients place financial value on test attributes, especially features that increase information available to patients, presents a challenge to practitioners and test sponsors alike.

Many inherited neuromuscular diseases share a similar pathophysiology marked by progressive muscle degeneration despite being caused by pathogenic variants in different genes [5]. Common elements of these disorders include weakness in muscle groups, fatigue, functional difficulties, pain, cardiac symptoms, and pulmonary symptoms [6]. Pompe disease is caused by pathogenic variants in the *GAA* gene, resulting in lysosomal acid α-glucosidase deficiency and accumulation of glycogen in muscle tissue [7]. Although Pompe disease has a variable rate of progression, in addition to muscle weakness, one of its earliest observed manifestations is respiratory muscle impairment that decreases vital capacity [8]. The presentation of respiratory insufficiency prior to loss of ambulation is a unique characteristic of Pompe disease when compared with other muscular dystrophy conditions [9]. Early treatment with recombinant human GAA protein is critical for improved outcomes through preservation of the muscle structure [10].

Genetic tests developed for diagnosis of Pompe disease have traditionally fallen into one of two categories: single-gene tests (SGTs) or neuromuscular disease panel tests (NMDPTs) [11]. Genetic tests that include the *GAA* gene have many uses, including further assessing infants with abnormal Pompe disease newborn screening test results, patients with clinically suspected Pompe disease, or patients with as-yet-unexplained neuromuscular symptoms, including proximal muscle weakness [12, 13]. Because the symptoms of late-onset Pompe disease are similar to those of many other conditions, genetic testing can be especially useful in such cases. Genetic testing through genotyping can quickly identify the hundreds of *GAA* pathogenic variants known to cause Pompe disease.

Given the advantages and complexities of diagnostic genetic testing, we need a better understanding of patients’ preferences for the features of different genetic tests that can be used for earlier diagnosis of rare diseases such as rare neuromuscular diseases. Discrete-choice experiments (DCE) can be used to elicit preferences for features of a good or service as revealed by choice among alternatives characterized by the different features of interest. Previously conducted DCEs for genomic sequencing to diagnose rare diseases have suggested variability in individuals’ perceived value of genetic tests for diagnosis, especially when considering test costs, time until a diagnosis, and availability of treatment [14–16].

Options for earlier diagnosis and subsequent treatment of rare diseases, such as neuromuscular diseases, could be improved by understanding the preferences of patients for the features of different genetic tests for these diseases. The objective of this study was to quantify patient preferences for features of genetic tests that could provide earlier diagnoses of rare neuromuscular diseases. We used the contrast between SGTs and NMDPTs for Pompe disease as the basis of our example, but the findings apply more broadly to the use of diagnostic genetic tests.

## Methods

### Study design

This study was granted a review exemption from the RTI International Institutional Review Board and followed good research practice guidelines published by the International Society for Pharmacoeconomics and Outcomes Research [17]. The draft survey instrument was pretested with a convenience sample of 15 participants recruited from the general population and then administered to an online panel with a target sample of the general population comprising 600 respondents. To be eligible for this survey, respondents were required to be aged 18 to 50 years and reside in the United States. The restriction of 50 years for age was in alignment with onset of symptoms of Pompe disease, which typically occur before age 50 [18].

### Survey instrument

The survey included screening questions, the obtaining of informed consent, and the collection of demographic information. For the DCE questions, six attributes with multiple levels were used to define the genetic tests (Table 1). Respondents were provided patient-friendly descriptions of the test attributes using plain language and asked background and comprehension questions about genetic tests (full descriptions of each attribute as provided to the respondents are presented in Table S-1, Supporting Information). The attributes and levels were based on the key components of SGT and NMDPT genetic tests available for Pompe disease at the time of study design and for the traditional muscle biopsy approach to diagnosis of Pompe disease [11]. The comparison between NMDPT and SGT allowed us to explore respondents’ perspectives on a number of key issues confronting the use of genetic testing generally.

The first attribute was the number of diseases tested for and the associated probability that the respondent would receive a diagnosis from that test. The survey included options for a single disease test and two panel tests for either 34 diseases or 100 diseases, which were included to represent tests for a broader set of rare genetic muscular conditions. For each test (single disease, panel of 34 diseases, and panel of 100 diseases), respondents were told the probability of a diagnosis as presented in Table 1. Time until diagnosis was thought to be an important attribute because it often takes many years to diagnose rare muscle diseases like Pompe disease. Two measures of time were included. The first measure of time was the time until diagnosis if the test described in the profile was positive. The second measure of time was the average time until a final diagnosis if the test described in the profile was negative. If a person has Pompe disease or another similar genetic disease, an SGT would provide a diagnosis more quickly. However, if someone starts with the SGT and does not have the disease that is the target of the SGT, the assumption is that the diagnosis would take longer than if he or she had started with a test for multiple diseases. The availability of treatment is another important consideration for genetic testing. The study team was interested in learning how the availability of a treatment affected respondent interest in the test and whether respondents placed value on getting a diagnosis in the absence of treatment.

The study team believed that some people are concerned about the possibility that the genetic test will contain information about the risk of unrelated health conditions, especially when the information is unclear or not actionable. Other individuals may be interested in learning as much as possible about the health risks they face, and these people may prefer a test that looks for pathogenic variants in a larger number of genes. The attribute measuring whether the test might include unrelated health information was included to explore this topic. Because muscle biopsy may be included in the diagnostic process for Pompe disease and other neuromuscular diseases, the team was interested in the relative importance of avoiding a muscle biopsy in choosing a genetic test.

The DCE section presented each respondent with a series of nine DCE choice questions following an experimental design generated in SAS using a D optimal algorithm to construct a fractional factorial experimental design [19–21]. The design comprised 54 DCE questions that were split into six blocks of nine questions each (an example of a choice question is presented in Figure S-1, Supporting Information). To minimize respondent fatigue, the full design was split into six blocks and each respondent was randomly assigned to answer nine questions from one of these blocks. Studies have suggested that respondents’ learning during the first few choice questions and respondents’ fatigue after answering many choice questions contribute to measurement error, and thus, affect preference estimates [22, 23]. To avoid having some questions systematically affected by learning and fatigue, we also randomized the order of the choice questions in each block for each respondent.

**Table 1:** Attributes and levels for the choice questions

Attribute	Level
Number of rare muscle diseases tested for (chance of getting a final diagnosis from the test)	100 diseases (60% chance) 34 diseases (30% chance) 1 disease (5% chance)
Treatment availability for diseases covered by the test^a^	Treatment is available for 100 diseases Treatment is available for 34 diseases Treatment is available for 1 disease Treatment is not available
Time until results from the test	1 month 2 months 6 months
Test results may contain information about unrelated health risks	Yes No
A muscle biopsy is needed to confirm results	Yes No
Average time until final diagnosis, if the first test is negative	2 years 4 years 7 years

^a^ The experimental design was created using three levels: treatment is available for all diseases tested for by the genetic test, treatment is available for one of the diseases tested for by the genetic test, and treatment is not available for any of the diseases tested for by the genetic test. The text in the survey instrument used the four levels listed in Table 1. For example, if the number of rare muscle diseases tested for was 34 and treatment availability for diseases covered by the test was “all,” respondents saw “Treatment is available for 34 diseases.”

**Fig. 1:**
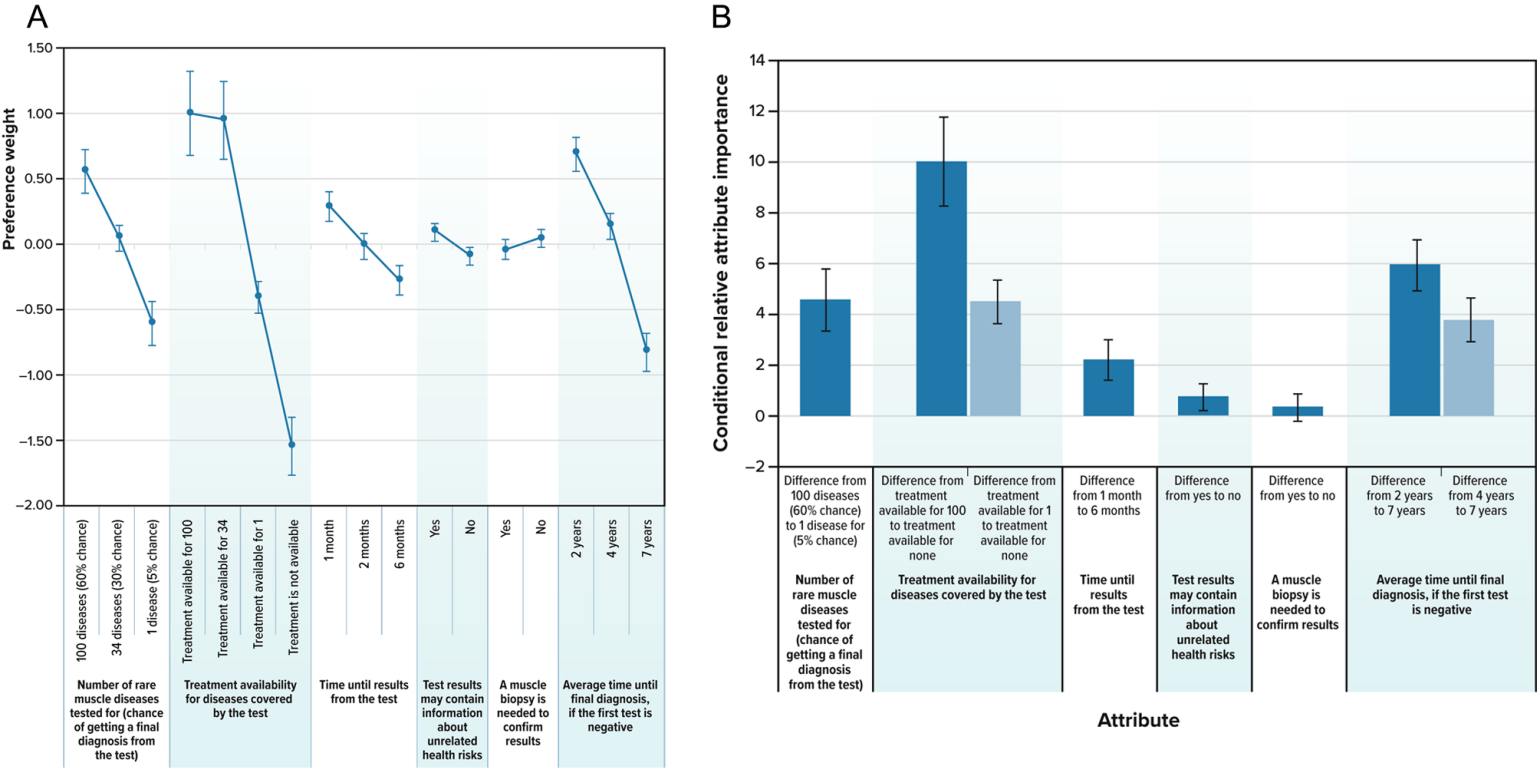
Preference weights (**A**) and conditional relative attribute importance (**B**) for overall respondents. ^a^The vertical bars surrounding each weight estimate indicate 95% confidence intervals

We were also interested in exploring the impact of cost on the choice between the tests. Cost was not included as an attribute in the DCE to avoid the possibility that respondents predominantly focused on cost, which would limit the information that could be learned about the other attributes [24, 25]. To provide some information on the impact of cost, after the sequence of DCE questions, respondents were asked in a direct elicitation question to choose between two test profiles in a choice question similar to the ones included in the DCE, but with cost included as an attribute. In this question, the first profile, Genetic Test A, represented test features similar to an SGT for Pompe disease, and the second, Genetic Test B, represented test features similar to a 34-disease NMDPT (Table 2). The cost of Genetic Test A was $40 and was modeled after an SGT. The cost of Genetic Test B, modeled after an NMDPT, was randomized across the sample to be associated with a cost of either $150 (*n* = 300) or $500 (*n* = 300).

We included measures based on the Ambiguity subscale of the Need for Closure Scale [26] and an adapted version of the Control Preferences Scale [27]. Measures such as these are of interest as predictors of preferences [28].

**Table 2:** Profiles for preference share prediction

Profiles for Cost Comparison		
Attribute	Genetic Test A	Genetic Test B
Number of rare muscle diseases tested for (chance of getting a final diagnosis from the test)	1 disease (5% chance)	34 diseases (30% chance)
Treatment availability for diseases covered by the test	Treatment is available for 1 disease	Treatment is available for 1 disease
Time until results from the test	2 months	6 months
Test results may contain information about unrelated health risks	No	No
A muscle biopsy is needed to confirm results	No	Yes
Average time until you get a final diagnosis, if the first test is negative	4 years	2 years
**Profiles for Number of Diseases Tested for and Availability of a Treatment**		
**Attribute**	**Genetic Test C**	**Genetic Test D**
Number of rare muscle diseases tested for (chance of getting a final diagnosis from the test)	1 disease (5% chance)	100 diseases (60% chance)
Treatment availability for diseases covered by the test	Treatment is available for 1 disease	Treatment is not available
Time until results from the test	2 months	2 months
Test results may contain information about unrelated health risks	No	No
A muscle biopsy is needed to confirm results	No	No
Average time until you get a final diagnosis, if the first test is negative	4 years	4 years

### Statistical analysis

Demographic data were summarized using descriptive statistics. The DCE data were analyzed using a random-parameters logit (RPL) regression model that relates the choices respondents make to the differences in the attribute levels across the alternatives in each choice question [29]. The RPL model avoids potential estimation bias from unobserved preference heterogeneity among respondents by estimating a distribution of preferences for each preference parameter [30, 31]. The preferences for all attribute levels were assumed to be normally distributed and simulated in estimation (using NLOGIT 5) using 1,000 Halton draws. All the levels in each attribute were effects coded (Table S-2, Supporting Information). We used a *t* test to determine the statistical significance of differences between adjacent attribute levels (*P* < 0.05) for each attribute. All estimates were reported with 95% confidence intervals.

The difference between the most-preferred and least-preferred levels of an attribute can be considered a measure of the conditional relative importance of that attribute—the importance of an attribute relative to the other attributes in the study given the range of levels included in the design. Relative importance was scaled such that the most important attribute was set to 10, and the conditional importance of each of the other attributes was scaled relative to this attribute. Preference differences were explored across the following seven subgroups: (1) willingness to get a genetic test that might return information about unrelated health conditions or not, (2) preference to make medical decisions themselves versus medical decisions with the help of a doctor, (3) score of “high” on the Need for Closure Ambiguity subscale, (4) score of “low” on the Need for Closure Ambiguity subscale, (5) respondents who had a 4-year college degree or higher versus less than a 4-year college degree, (6) gender, and (7) previous experience with genetic tests. For each mutually exclusive set of subgroups, we created a dummy variable that was equal to 1 if the respondent belonged to the subgroup and interacted the dummy variable with each of the attribute levels. Differences in preferences between subgroups were tested through a Wald ${\chi }^{2}$ test of joint statistical significance of all the interaction terms (*P* < 0.05).

To explore the impact of cost on choice, predicted-choice probabilities were computed for the profiles in Table 2 when cost was not included. More specifically, the predicted-choice probability was calculated as the probability that the average respondent selects one treatment over another, using the mean RPL model estimates. The predicted probability that the average respondent would choose Genetic Test A and Genetic Test B from Table 2 without cost was compared with the proportion of respondents in the sample who selected each test in these direct elicitation questions that included cost as an attribute. In addition, to explore the importance of treatment availability, we used the RPL model estimates to create preference shares for a genetic test for 1 disease with treatment available and a genetic test for 100 diseases with no treatments available, holding all else constant (Table 2).

## Results

Six hundred respondents who met the eligibility criteria and provided informed consent were recruited via email from the Lightspeed online panel and partner panels. Demographic information is presented in Table 3. The majority of respondents (80%) identified as white, 10.7% identified as black or African American, and 10% described their ethnicity as Hispanic or Latino. Almost half (42%) of respondents reported a household income of less than $50,000, whereas 53.5% reported a household income of $50,000 or more. The average age of respondents was 36.6 years, and 51.5% of the sample were female. The majority of the sample (86.7%) reported being familiar with genetic tests, with one-quarter (24.8%) of the sample reporting having had a genetic test (Table S-3, Supporting Information). The most common genetic tests reported were screening tests and tests to learn about ancestry. The majority of respondents agreed with statements that indicated not liking uncertainty or ambiguity. For example, 87.3% of respondents agreed with the statement “I don’t like situations that are uncertain.” The majority of respondents (66.9%) preferred making their own medical decisions about treatment either by themselves or after seriously considering their doctor’s opinion versus sharing the decision with their doctor (26.2%) or letting their doctor make the decision (7.0%) (Table S-4, Supporting Information).

**Table 3:** Demographic characteristics

Question	All Respondents *N* = 600^a^
**Mean age ± SD in years**	36.6 ± 10.17
**Gender**	
n	600
Female	309 (51.5%)
Male	288 (48.0%)
Other or prefer not to say	3 (0.5%)
**Marital status**	
Single/never married/other	260 (43.3%)
Married/living as married/civil partnership	288 (48.0%)
Divorced or separated	52 (8.7%)
**Highest level of education**	
High school or equivalent or less	114 (19.0%)
Some college but no degree	142 (23.7%)
Technical school or Associate’s degree	82 (13.7%)
4-year college degree	162 (27.0%)
Graduate or professional school	100 (16.7%)
**Employment status**	
Employed	402 (67.0%)
Homemaker	52 (8.7%)
Student	58 (9.7%)
Otherwise not working	88 (14.7%)
**Total household income before tax and other deductions**	
Less than $50,000	253 (42.2%)
$50,000 to $74,999	122 (20.3%)
Over $75,000	199 (33.2%)
**Race**	
American Indian or Alaska Native	11 (1.8%)
Asian Indian	13 (2.2%)
Black or African American	64 (10.7%)
Chinese	11 (1.8%)
Filipino	4 (0.7%)
Japanese	8 (1.3%)
Korean	1 (0.2%)
Other Asian	5 (0.8%)
Native Hawaiian or other Pacific Islander	1 (0.2%)
White	477 (79.5%)
Other or prefer not to share	31 (5.2%)
**Ethnicity**	
Hispanic or Latino	60 (10.0%)
Not Hispanic or Latino	528 (88.0%)
Prefer not to share	12 (2.0%)

^a^ May not total 100% for each category. SD = standard deviation

### DCE Preference

Respondents preferred tests that checked for more diseases and had a higher probability of providing a diagnosis, tests that checked for diseases with an available treatment, tests that provided results more quickly, tests that might provide information about unrelated health risks, and tests with a sequence that provided the final diagnosis sooner (Fig. 1A). Whether the test required a muscle biopsy was not a factor in respondent choices, suggesting that the need for a muscle biopsy is relatively less important than changes in the other test attributes. The remaining levels within each attribute were statistically significantly different from one another (*P* < 0.05), except for the difference between testing for 100 or 34 diseases. Over the ranges presented in the survey, the relative importances of the largest change in each attribute from most to least important are presented in Fig. 1A. In addition, having treatment available for at least one of the diseases included in the genetic test compared to having no treatment was as important as the difference between a test for 1 disease with a 5% probability of diagnosis and a test for 100 diseases with a 60% probability of diagnosis. Finally, when considering the average time until diagnosis if the first test is negative, the difference between waiting 4 years and 7 years accounted for much of the overall importance of the attribute, suggesting that the 3 additional years of waiting for a diagnosis between 4 years and 7 years has higher disutility than the 2 years of waiting between 2 years and 4 years.

We investigated seven different subgroups to explore explained preference heterogeneity. Only the subgroups based on gender and the respondent’s willingness to get a test that might reveal information about unrelated health risks resulted in statistically significant systematic differences in preferences at the 5% statistical significance level (Table 4). Women had statistically significantly different preferences compared with the rest of the sample (*P* = 0.046) (Figure S-2, Supporting Information). Both men and women preferred a test that included more diseases with a higher probability of diagnosis, and women placed a higher relative importance on this attribute compared to the other attributes than the rest of the sample. Women preferred that the test have the possibility of providing unrelated health information, whereas, for men, the two levels of the attribute were not statistically significantly different from each other.

**Table 4:** Exploratory subgroup analysis

Subgroup Pair	Subgroup Description	n	*P* Value
Respondents who were willing to get a genetic test that might return information about unrelated health conditions versus those who were not^a^	Respondents who were willing to get a genetic test that might return information about unrelated health conditions	491	< 0.000
	Respondents who were not willing to get a genetic test that might return information about unrelated health conditions	109	
Respondents who preferred to make medical decisions themselves versus those who preferred to make medical decisions with the help of a doctor versus those who did not	Respondents who preferred to make medical decisions themselves versus those who preferred to make medical decisions with the help of a doctor	401	0.202
	Respondents who did not prefer to make medical decisions themselves versus those who preferred to make medical decisions with the help of a doctor	199	
Respondents who scored “high” on the Need for Closure Ambiguity subscale [26] versus those who did not	High score	173	0.326
	All others	427	
Respondents who scored “low” on the Need for Closure Ambiguity subscale versus [26] those who did not	Low score	183	0.077
	All others	417	
Respondents who have a 4-year college degree or more versus those who do not	4-year college degree or more	262	0.739
	Less than a 4-year college degree	338	
Respondents who are female versus those who are not	Female	309	0.046
	All others	291	
Respondents who have had experience with genetic tests versus those who had not	Experience with genetic tests	149	0.742
	No experience with genetic tests	451	

^a^ This model was estimated only interacting the subgroup with the unrelated health-risk attribute

**Fig. 2:**
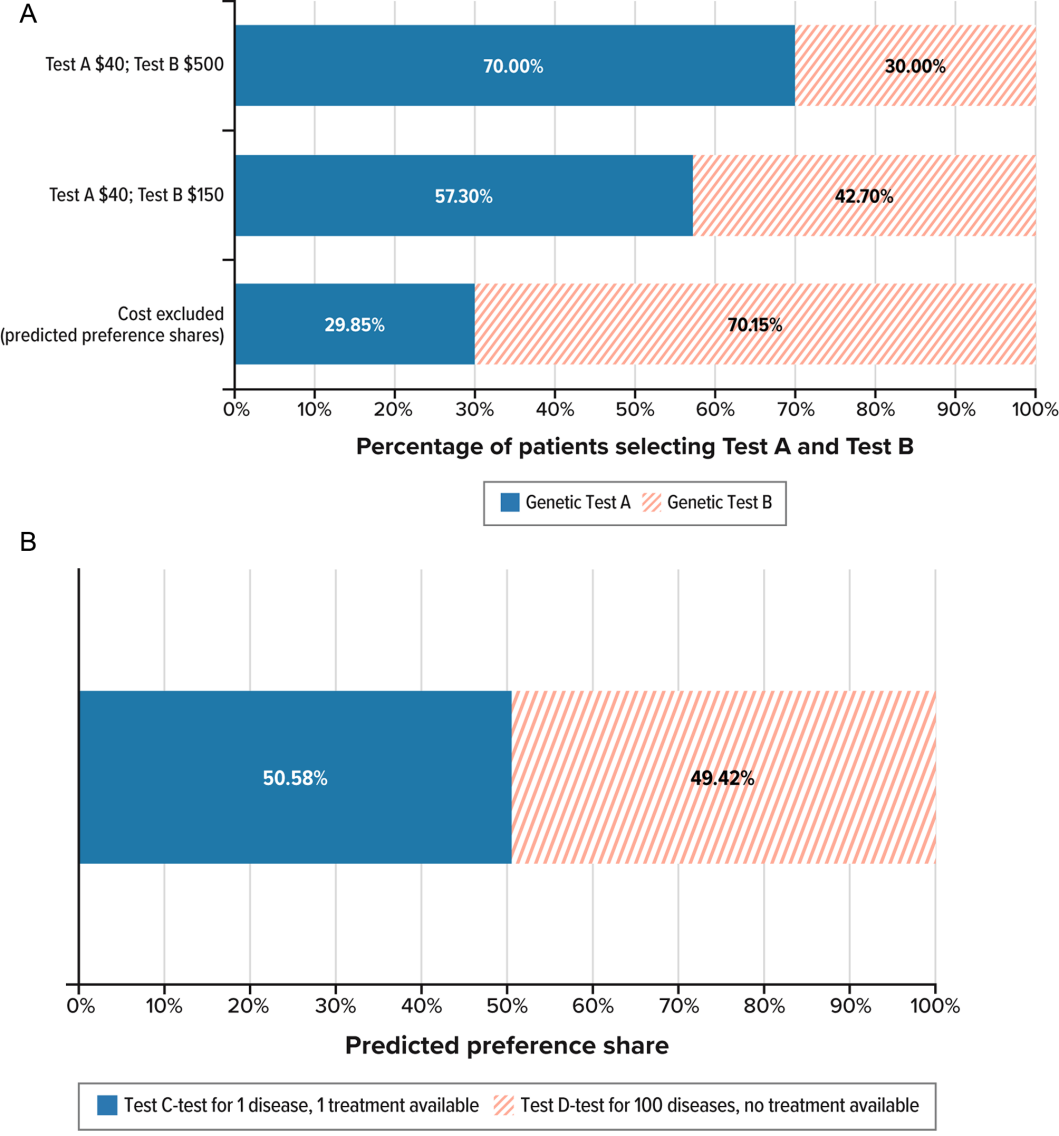
Probability of selecting each test. (**A**) Change in probability that respondents selected each test when cost was included. (**B**) Probability of selecting a test for 1 disease with a treatment over a test for 100 diseases with no treatments.

The subgroup for interest in information about unrelated health conditions is based on the question, “If a medical test might find information about health risks that are unrelated to the condition you are testing for, how would that affect your willingness to get the test?” A total of 109 respondents said that they would be much less or somewhat less willing to get the test, while 491 respondents said they were indifferent, somewhat more willing, or much more willing to get the test or were unsure about getting the test. The group that responded that they would be much less willing to get the test that might return unrelated health-risk information preferred a test that did not include the possibility of information about unrelated health risks when the health-risk subgroup interacted with the attribute “test results may contain information about unrelated health risks” (Figure S-3, Supporting Information). In contrast, the respondents who were indifferent, somewhat more willing, or much more willing to get the test or were unsure about getting the test preferred a test that might provide information about unrelated health risks.

Additionally, the subgroup results comparing respondents who scored “low” on the Need for Closure Ambiguity subscale and those who did not trended toward significance (*P* = 0.077) (Figure S-4, Supporting Information). Both groups preferred testing for more diseases, getting test results faster, and getting a final diagnosis sooner if the first test is negative; neither group differentiated between needing a muscle biopsy or not. Most notably, while both groups preferred getting a final diagnosis sooner, those who scored low on the Need for Closure Ambiguity Scale placed a lower relative importance on average time until final diagnosis compared with the rest of the sample (Figure S-5, Supporting Information).

### Predicted-choice probabilities

Figure 2A compares the proportions of people who selected Genetic Test A and Genetic Test B, modeled after the SGT and NMDPT, in the direct elicitation question that included cost and the profiles described in Table 2. The preference shares for the two profiles were predicted using the mean RPL model estimates (based on the series of DCE questions without cost). When asked to select between Genetic Test A (the SGT for 1 disease) and Genetic Test B (the 34-disease panel) in the question including cost, 43% of respondents selected Genetic Test B when this test cost $150, while 30% of respondents selected Genetic Test B when this test cost $500. When the actual percentage of respondents who selected each test is compared with the predicted probability of selecting Genetic Tests A and B without cost using the RPL model results, the average respondent in the sample has a 70% probability of selecting Genetic Test B, suggesting that cost may factor into the choice between the tests.

In addition, we used the RPL model estimates to predict the probability that the average respondent would select a test for 1 disease (with a 5% probability of diagnosis) that has a treatment available compared with a test for 100 diseases (with a 60% probability of diagnosis) and no treatments available (Genetic Test C and Genetic Test D, respectively). Figure 2B shows these predicted preference shares for the profiles in Table 2. The model predicted that the average respondent in the sample has approximately a 50% chance of selecting either Genetic Test C or Genetic Test D. On average, testing for 1 disease with one available treatment and a 5% probability of a diagnosis was viewed as about equal in utility to testing for 100 diseases with no treatments available and a 60% probability of diagnosis.

## Discussion

We evaluated the preferences of adults for genetic tests used to diagnose rare muscle diseases by using a set of test features that included the number of diseases tested for, the probability of diagnosis, treatment availability, time until test results received, whether test results may contain information about unrelated health risks, whether a muscle biopsy is needed to confirm results, and average time until final diagnosis. Respondents placed greatest importance on whether a treatment was available for diseases covered by the test and test attributes that decreased the duration of the diagnostic journey, including the number of diseases tested for, the probability of getting a diagnosis from the test, and the time to get a final diagnosis. On average, respondents preferred a test that might also return information on unrelated health risks. The recent advance from SGTs to panel tests capable of providing more information aligns with our study results that patients find value in knowing the probability of diagnosis, even for conditions without available treatments, and in knowing incidental findings.

While panel testing may have a lower chance of incidental findings than does clinical exome sequencing, incidental findings from panel testing can still provide knowledge valued by patients [32]. Other studies that included an attribute for return of incidental findings have similarly observed a preference for additional information from the test, as well as heterogeneity in respondents’ preferences [14, 33–35]. The degree of preference for the return of incidental or secondary findings varied among the respondents in Regier, Peacock [33] and Goranitis, Best [14], indicating some respondents found other test attributes to be more important. Ploug and Holm [34] also noted that the preference of patients for information increased with the risk of the disorder; however, there was still variation among respondents in their preferences for receiving information about unrelated findings. While the disclosure of high-risk information could increase patient distress, Lewis, Stine [35] observed information likely to cause distress was also likely to be information valued by patients. Our findings echo the literature in suggesting that the disclosure of potentially distressing information may need to be considered on an individual basis so as not to further contribute to the burden experienced by patients.

The diagnostic journey for rare diseases can be long and frustrating, and the results of our study emphasize the importance of getting a diagnosis sooner. Waiting a few more months for results from the first genetic test was not as important as reducing the number of years to a final diagnosis if the first test was negative. Additionally, tests for diseases with a treatment available were highly valued over tests for diseases without treatment. While this preference seems intuitive, the intensity of this preference revealed through quantification of the value was unexpected. The SGT for 1 disease with a 5% probability of diagnosis had equal utility to a test for 100 diseases with a 60% probability of diagnosis if the SGT had a treatment available and the test for 100 diseases had no treatments available for the diseases covered by the test. Although tests for diseases with treatments had high relative importance, respondents were still willing to trade off the availability of a treatment for improvements in other attributes, including time and the probability of a diagnosis. Because the survey did not offer the option of not getting a test, we do not know the value of getting a test for a disease with no treatment over not getting a test and having no diagnosis.

Some differences in preferences were found across the sample. The majority of respondents reported that they would be more likely to get a test that might provide information on unrelated health risks, but some respondents reported receiving information about unrelated health risks would make them less likely to get the test. The respondents who did not want secondary findings disclosed further corroborated this stance in the DCE section by preferring tests that did not reveal unrelated health risks. The differences among stated preferences underscores the importance of an individual, patient-centered approach to revealing secondary findings. We attempted to explain this preference heterogeneity through exploring seven different subgroups. We found significant differences based on gender and on the Need for Closure Ambiguity Scale. Women placed relatively more importance on a test for more diseases that had a higher probability of diagnosis than the rest of the sample. Respondents who scored lower on the Need for Closure Ambiguity Scale placed lower importance on time until diagnosis relative to the other attributes than did the rest of the sample.

Additionally, the direct elicitation question revealed that cost may strongly influence the choice between possible genetic tests. Panel testing in particular has the ability to substantially reduce the cost of diagnosing neuromuscular diseases for patients. Schofield, Alam [36] found the cost per diagnosis of a neuromuscular disease was $3,706 using a genetic panel compared with $16,495 per diagnosis for a traditional invasive biopsy. Paganoni, Nicholson [3] noted that genetic testing could improve the quality and value of care for neuromuscular diseases, especially in reducing the delay until diagnosis, and that value would likely continue to increase as the cost of testing decreases. Given the impact of test value and cost-effectiveness on utilization, it is not surprising that SGTs have been largely replaced by panel tests.

The results of the DCE survey should be interpreted in the context of limitations related to the survey instrument and sample. Creating a DCE survey instrument requires balancing a thorough description of the object of choice, in this case a genetic test, against the limits of respondent comprehension and burden. In the descriptions of the attributes and types of tests, efforts were made to present neutral descriptions that provided an accurate, concise description of the attributes. In particular, it was a challenge communicating the difference between the attributes for time until a diagnosis when the first test was positive and the average number of years until diagnosis if the first test was negative. Changes were made during the pretest to reinforce the interpretation of the attributes, including adding two comprehension questions about these attributes. Furthermore, the sample for the study was drawn from the general population, and respondents were asked to imagine that they had unexplained muscle weakness. It is possible that respondents who had experience with the diagnosis process for muscle weakness might have different relative preferences. The sample for this study valued faster diagnosis, and time until diagnosis is a frustration for patients with rare diseases [37]. Additionally, the survey presented hypothetical scenarios to respondents that did not replicate the experience of talking with a doctor about testing options. Decisions made in the survey may not fully predict decisions made in a clinical setting where other considerations may come into play. For example, in a real-world setting, clinicians use patients’ disease features (e.g., signs, symptoms, lab tests) to guide diagnoses and to determine the most appropriate type of genetic testing [38]. In addition, genetic testing is free for some rare diseases in certain European countries, which could influence the clinical decision-making process [39]. It should be noted that the samples in this study were convenience samples recruited through opt-in panels of individuals who chose to participate in research. Although the sample recruitment included quotas for gender, the study samples may not be representative of the broader population of adults aged 18 to 50 years. Lastly, the final survey was administered online. Research has shown that results from online stated-preference surveys are, in general, not statistically significantly different from those elicited through face-to-face interviews [40, 41]. However, the online setting of the survey may also have influenced the choices respondents made.

## Conclusion

Overall, respondents preferred tests that tested for more diseases and had a higher probability of diagnosis, tests that tested for diseases that had treatments available, and tests that led to a final diagnosis sooner. These preference study results highlight the important role genetic tests have in providing knowledge valued by patients. The preference we found for cost-effective genetic tests that can significantly reduce the time until diagnosis aligns with the current trend of using panel tests instead of tests for single genes. Importantly, the decrease in time to diagnose can reduce the duration of the diagnostic journey and its associated burdens on patients and their families.

### Electronic supplementary material

Below is the link to the electronic supplementary material.

Supplementary Material 1

## Data Availability

The data that support the findings of this study are available from the corresponding author upon reasonable request. Please contact Susan Sparks (Susan.Sparks@sanofi.com).
