# Supplementary material for: The value of knowing: preferences for genetic testing to diagnose rare muscle diseases
Source: Orphanet J Rare Dis. 2024 Apr 22;19:173. doi: 10.1186/s13023-024-03160-7 (PMC11036564; doi:10.1186/s13023-024-03160-7)
Supplement: Supplementary file 1 — Supplementary Material 1 [file 13023_2024_3160_MOESM1_ESM.docx]

# Supporting Information

Table S-1. Full Descriptions of Each Attribute Provided to the Respondents

| Attribute | Description |
| --- | --- |
| Number of rare muscle diseases tested for (chance of getting a final diagnosis from the test) | Genetic tests can look at one specific gene to test for one rare muscle disease or at a set of genes to test for a set of diseases. When you get a genetic test, the doctor takes a sample of your DNA. The doctor will get the DNA sample by using a needle to take blood from a vein in your arm or by getting a sample of your saliva or by brushing the inside of your cheek to get a sample of cells. The chance that you get a final diagnosis from the first genetic test you take will depend on the number of rare muscle diseases that are included in the genetic test. |
| Treatment availability for diseases covered by the test | Treatment is available for some rare muscle diseases, but not for others. In this survey, we ask you to assume that, if a treatment for a specific disease is available, it will stop the disease from getting worse, so it is important to start the treatment as early as possible. Even if there are no treatments for the diseases that the test can identify, there may be other benefits to getting an early diagnosis. Some benefits of an early diagnosis are as follows: knowing the cause of your muscle weakness so you don’t have to get more tests, giving your doctor more information so they can better monitor your condition, or using the information about your genes for family planning. |
| Time until results from the test | Some genetic tests can be analyzed faster than others. If you test positive for one of the diseases included in the first genetic test you take, you might get your diagnosis faster. If the first genetic test is negative, you will need follow-up tests, and it will take longer to get a final diagnosis.  Later in the survey, we will ask you to consider genetic tests that could provide results in 1 month to 6 months. |
| Test results may contain information about unrelated health risks | When you get a genetic test for diseases that cause muscle weakness, there is a chance that the test results will contain information about health risks that are not related to muscle weakness. For example, you might find out you are at higher risk of developing a certain type of cancer or another disease in the future.  Sometimes the extra information is useful, but sometimes the test results are not clear or you can test positive for a potential health problem that you will never develop. |
| A muscle biopsy is needed to confirm results | Before genetic testing was widely used, doctors used muscle biopsy and other tests to test for rare muscle diseases. Even with genetic testing, a muscle biopsy may still be needed to confirm the results of the genetic test.  The following will occur if you get a muscle biopsy   - The doctor will numb the site by injecting you with a local anesthetic - The doctor will take a sample of muscle tissue using a large needle or by cutting away a piece of muscle - You may need stiches - You will have a small scar, and the area will be tender and sore for a couple of days |
| Average time until final diagnosis, if the first test is negative | If the genetic test you select to start with is negative (you don’t have the diseases covered by the test), you will need to get tested for more diseases to get a diagnosis. Different tests provide different information, and the average amount of time until your disease is diagnosed if the first test is negative will depend on the results from the first test and what makes the most sense for the next tests.  Later in the survey, we will ask you to choose between options that take different amounts of time on average before you get a final diagnosis if the first test you get is negative. The range of time is based on the experience of other patients diagnosed with rare diseases that cause muscle weakness. The average time until you get a final diagnosis will vary from 2 years to 7 years if the first test you get is negative. Please note that if the first test you get is negative and you are tested for additional diseases, we don’t know if there is treatment available for the final disease you are diagnosed with. |

Table S-2. Random-Parameters Logit Model Variables for Estimating Preference Weights

| Variable Label | Variable Definition |
| --- | --- |
| TEST_100  TEST_34  TEST_1 | Effects-coded variables for number of rare muscle diseases tested for (TEST_100 = 100 diseases [60% chance]; TEST_34 = 34 diseases [30% chance]; TEST_1 = 1 disease [5% chance]). TEST_1 was omitted for model identification. |
| TREA_100  TREA_34  TREA_1  TREA_N | Effects-coded variable for treatment available for diseases covered by the test (TREA_100 = Treatment available for 100 diseases; TREA_34 = Treatment available for 34 diseases; TREA_1 = Treatment available for 1 disease; TREA_N = Treatment is not available). TREA_N was omitted for model identification. |
| TTIME_1  TTIME_2  TTIME_6 | Effects-coded variable for time until you get results from the test (TTIME_1 = 1 month; TTIME_2 = 2 months; TTIME_6 = 6 months). TTIME_6 was omitted for model identification. |
| RISK_Y  RISK_N | Effects-coded variable for test results may contain information about unrelated health risks (RISK_Y = Yes; RISK_N = No). RISK_N was omitted for model identification. |
| BIOP_N  BIOP_Y | Effects-coded variable for a muscle biopsy is needed to confirm results (BIOP_Y = Yes; BIOP_N = No). BIOP_N was omitted for model identification. |
| ATIM_2  ATIM_4  ATIM_7 | Effects-coded variable for if the first test is negative: average time until you get a final diagnosis after testing for more diseases (ATIM_2 = 2 years; ATIM_4 = 4 years; ATIM_7 = 7 years). ATIM_7 was omitted for model identification. |

Table S-3. Experience With Genetic Tests and Muscle Weakness

| **Question** | All Respondents N = 600 |
| --- | --- |
| All respondents | |
| Had you ever heard of genetic testing before reading the information above? |  |
| Yes | 520 (86.7%) |
| No | 63 (10.5%) |
| Don't know or not sure | 17 (2.8%) |
| Have you ever heard about newborn screening programs to test for the risk of childhood diseases? |  |
| Yes | 441 (73.5%) |
| No | 117 (19.5%) |
| Don't know or not sure | 42 (7.0%) |
| Have you ever had to get multiple tests to diagnose a serious health problem, either genetic tests or other types of tests? |  |
| Yes | 220 (36.7%) |
| No | 349 (58.2%) |
| Don't know or not sure | 31 (5.2%) |
| Do you currently have any symptoms of muscle weakness? |  |
| Yes | 84 (14.0%) |
| No | 491 (81.8%) |
| Don't know or not sure | 25 (4.2%) |
| Have you ever gone to the doctor because of muscle weakness that was getting worse over time? |  |
| Yes | 68 (11.3%) |
| No | 525 (87.5%) |
| Don't know or not sure | 7 (1.2%) |
| Among respondents who have gone to the doctor because of muscle weakness | |
| How severe were your symptoms when you went to see the doctor? |  |
| n | 68 |
| Very mild, did not interfere with my daily life at all | 15 (22.1%) |
| Mild, interfered only slightly with my usual activities | 30 (44.1%) |
| Moderate, interfered with my ability to do my usual work, school, or social activities | 18 (26.5%) |
| Severe, I needed help with activities like bathing, dressing, and feeding myself | 5 (7.4%) |
| Was your doctor able to diagnose the cause of the muscle weakness? | |
| n | 68 |
| Yes | 43 (63.2%) |
| No | 20 (29.4%) |
| Don't know or not sure | 5 (7.4%) |
| Among respondents whose muscle weakness has been diagnosed | |
| Have you ever been diagnosed with any of the following diseases? Please check all that apply.^a^ |  |
| n | 43 |
| Pompe disease | 3 (7.0%) |
| Muscular dystrophy (limb girdle, FSH) | 9 (20.9%) |
| Duchenne/Becker muscular dystrophy | 2 (4.7%) |
| Spinal muscular atrophy, any type | 7 (16.3%) |
| Myotonic dystrophy | 2 (4.7%) |
| Myasthenia gravis | 2 (4.7%) |
| Glycogen storage disease | 1 (2.3%) |
| Polymyositis, any type | 2 (4.7%) |
| Inclusion body myositis | 2 (4.7%) |
| Myopathy, any type | 2 (4.7%) |
| Mitochondrial metabolism disorders | 4 (9.3%) |
| MELAS syndrome | 1 (2.3%) |
| MERRF syndrome | 0 |
| Other | 11 (25.6%) |
| None of the above | 11 (25.6%) |
| All respondents | |
| Have you ever had a genetic test of any kind, including genetic screening tests, genetic tests to diagnose a disease or identify a health risk, or a genetic test to learn about your family ancestry? |  |
| Yes | 149 (24.8%) |
| No | 424 (70.7%) |
| Don't know or not sure | 27 (4.5%) |
| Among respondents who have had a genetic test | |
| What types of genetic tests have you had? Please check all that apply.^a^ |  |
| n | 149 |
| Screening test looking for the risk of future health problems | 64 (43.0%) |
| Screening test when pregnant looking for the risk of health problems for the baby | 34 (22.8%) |
| Diagnostic test to help find a diagnosis for a specific set of symptoms you were having | 54 (36.2%) |
| Diagnostic test to help pick the best treatment for a disease that you had | 22 (14.8%) |
| Genetic test to learn about family ancestry | 66 (44.3%) |
| Other | 11 (7.4%) |
| Don't know or not sure | 13 (8.7%) |

^a^ May not total 100% for each category.

Table S-4. Responses to Patient-Reported Outcome Instruments

| Question | All Respondents N = 600 |
| --- | --- |
| **All respondents** | |
| I don't like situations that are uncertain |  |
| n | 600 |
| Strongly disagree | 18 (3.0%) |
| Moderately disagree | 17 (2.8%) |
| Slightly disagree | 41 (6.8%) |
| Slightly agree | 166 (27.7%) |
| Moderately agree | 182 (30.3%) |
| Strongly agree | 176 (29.3%) |
| I feel uncomfortable when I don't understand the reason why an event occurred in my life |  |
| n | 600 |
| Strongly disagree | 14 (2.3%) |
| Moderately disagree | 26 (4.3%) |
| Slightly disagree | 62 (10.3%) |
| Slightly agree | 152 (25.3%) |
| Moderately agree | 188 (31.3%) |
| Strongly agree | 158 (26.3%) |
| When I am confused about an important issue, I feel very upset |  |
| n | 600 |
| Strongly disagree | 20 (3.3%) |
| Moderately disagree | 36 (6.0%) |
| Slightly disagree | 94 (15.7%) |
| Slightly agree | 175 (29.2%) |
| Moderately agree | 152 (25.3%) |
| Strongly agree | 123 (20.5%) |
| In most social conflicts, I can easily see which side is right and which is wrong |  |
| n | 600 |
| Strongly disagree | 10 (1.7%) |
| Moderately disagree | 11 (1.8%) |
| Slightly disagree | 63 (10.5%) |
| Slightly agree | 195 (32.5%) |
| Moderately agree | 209 (34.8%) |
| Strongly agree | 112 (18.7%) |
| I like to know what people are thinking all the time |  |
| n | 600 |
| Strongly disagree | 37 (6.2%) |
| Moderately disagree | 48 (8.0%) |
| Slightly disagree | 122 (20.3%) |
| Slightly agree | 182 (30.3%) |
| Moderately agree | 134 (22.3%) |
| Strongly agree | 77 (12.8%) |
| I dislike it when a person's statement could mean many different things |  |
| n | 600 |
| Strongly disagree | 18 (3.0%) |
| Moderately disagree | 27 (4.5%) |
| Slightly disagree | 85 (14.2%) |
| Slightly agree | 225 (37.5%) |
| Moderately agree | 121 (20.2%) |
| Strongly agree | 124 (20.7%) |
| It's annoying to listen to someone who cannot seem to make up his or her mind |  |
| n | 600 |
| Strongly disagree | 14 (2.3%) |
| Moderately disagree | 19 (3.2%) |
| Slightly disagree | 72 (12.0%) |
| Slightly agree | 171 (28.5%) |
| Moderately agree | 155 (25.8%) |
| Strongly agree | 169 (28.2%) |
| I feel uncomfortable when someone's meaning or intention is unclear to me |  |
| n | 600 |
| Strongly disagree | 15 (2.5%) |
| Moderately disagree | 19 (3.2%) |
| Slightly disagree | 66 (11.0%) |
| Slightly agree | 182 (30.3%) |
| Moderately agree | 188 (31.3%) |
| Strongly agree | 130 (21.7%) |
| I'd rather know bad news than stay in a state of uncertainty |  |
| n | 600 |
| Strongly disagree | 10 (1.7%) |
| Moderately disagree | 10 (1.7%) |
| Slightly disagree | 37 (6.2%) |
| Slightly agree | 139 (23.2%) |
| Moderately agree | 167 (27.8%) |
| Strongly agree | 237 (39.5%) |
| Ambiguity subscale of the Need for Closure Scale Score^a^ |  |
| n | 600 |
| Mean (SD) | 40.3 (7.40) |
| Median | 41.0 |
| Q1, Q3 | 36.0, 45.0 |
| Min, max | 9, 54 |
| Which of the following statements about making medical decisions would you say describes you best? |  |
| n | 600 |
| I prefer to make the decision about which treatment I will receive | 142 (23.7%) |
| I prefer to make the final decision about my treatment after seriously considering my doctor's opinion | 259 (43.2%) |
| I prefer that my doctor and I share responsibility for deciding which treatment is best for me | 157 (26.2%) |
| I prefer that my doctor makes the final decision about which treatment will be used, but seriously considers my opinion | 31 (5.2%) |
| I prefer to leave all decisions regarding treatment to my doctor | 11 (1.8%) |

Q1 = first quartile; Q3 = third quartile; SD = standard deviation.

^a^ The Ambiguity subscale is calculated using the rules from [Kruglanski et al. (2013)](#_ENREF_12). The top and bottom quartiles are used to determine high and low ambiguity.

Figure S-1. Example of Choice Question

| **Test Feature** | **Start With Genetic Test A** | **Start With Genetic Test B** |
| --- | --- | --- |
| **Number of rare muscle diseases tested for** | 1 disease | 34 diseases |
| **Chance you get a final diagnosis from the test** | 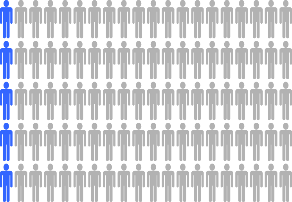  5 out of 100 people (5%) | 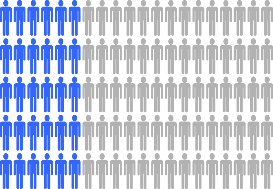  30 out of 100 people (30%) |
| **Time until you get results from the test** | 1 month | 6 months |
| **Treatment available for diseases covered by the test** | Treatment is available for 1 disease | Treatment is available for 34 diseases |
| **Test results may contain information about unrelated health risks** | No | Yes |
| **A muscle biopsy is needed to confirm results** | Yes | No |
| **If the first test is negative: Average time until you get a final diagnosis after testing for more diseases** | **2 years**  and unknown if treatment available for the final diagnosis  (will be true for 95% of people who did not get a diagnosis from the first test) | **4 years**  and unknown if treatment available for the final diagnosis  (will be true for 70% of people who did not get a diagnosis from the first test) |
| **Which test would you choose?** |  |  |

Figure S-2. Preference Weights Subgroup Analysis Based on Gender


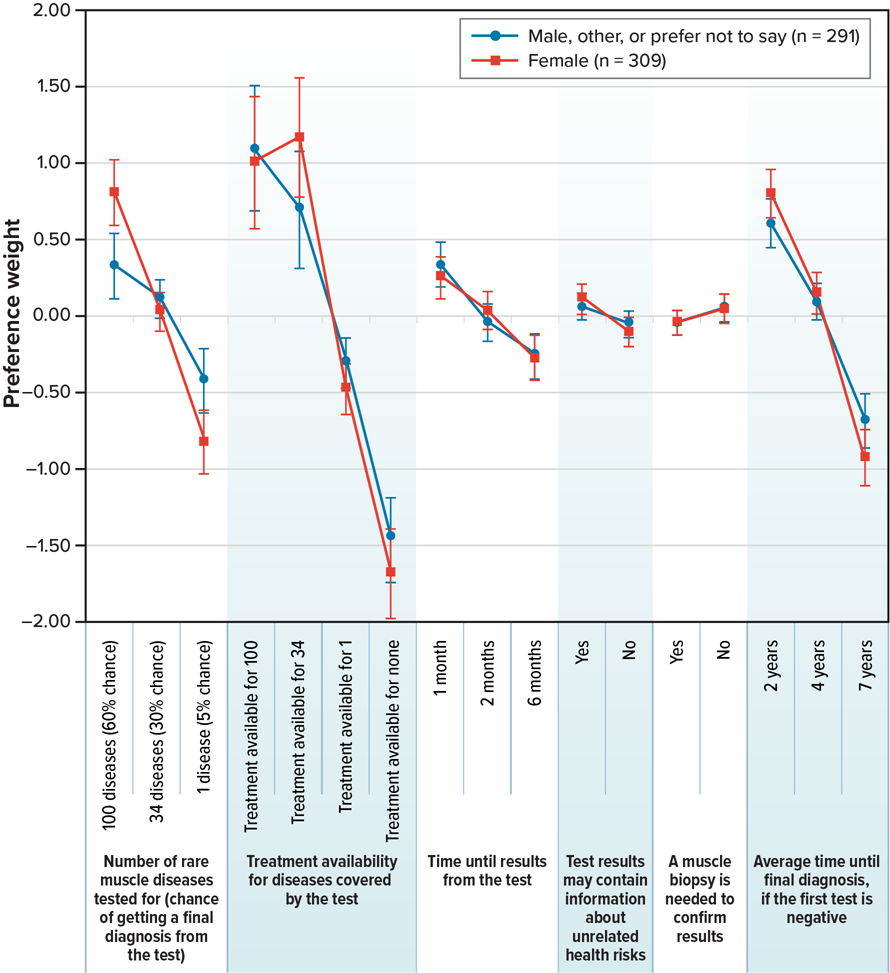


Note: The vertical bars surrounding each mean preference weight denote the 95% confidence interval of the point estimate.

Figure S-3. Preference Weights Subgroup Analysis Based on Whether Respondent Was Interested in Information About Unrelated Health Risks


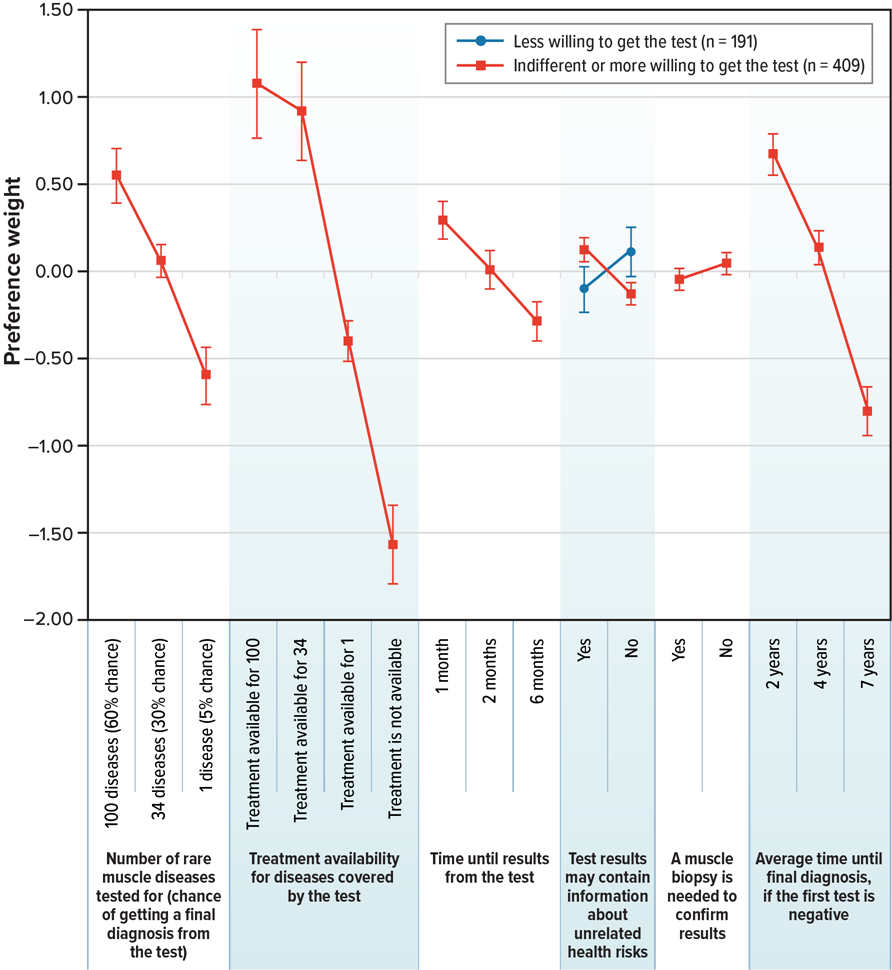


Figure S-4. Preference Weights Subgroup Analysis Based on Whether Respondent Scored Low on the Need for Closure Ambiguity Scale or Not


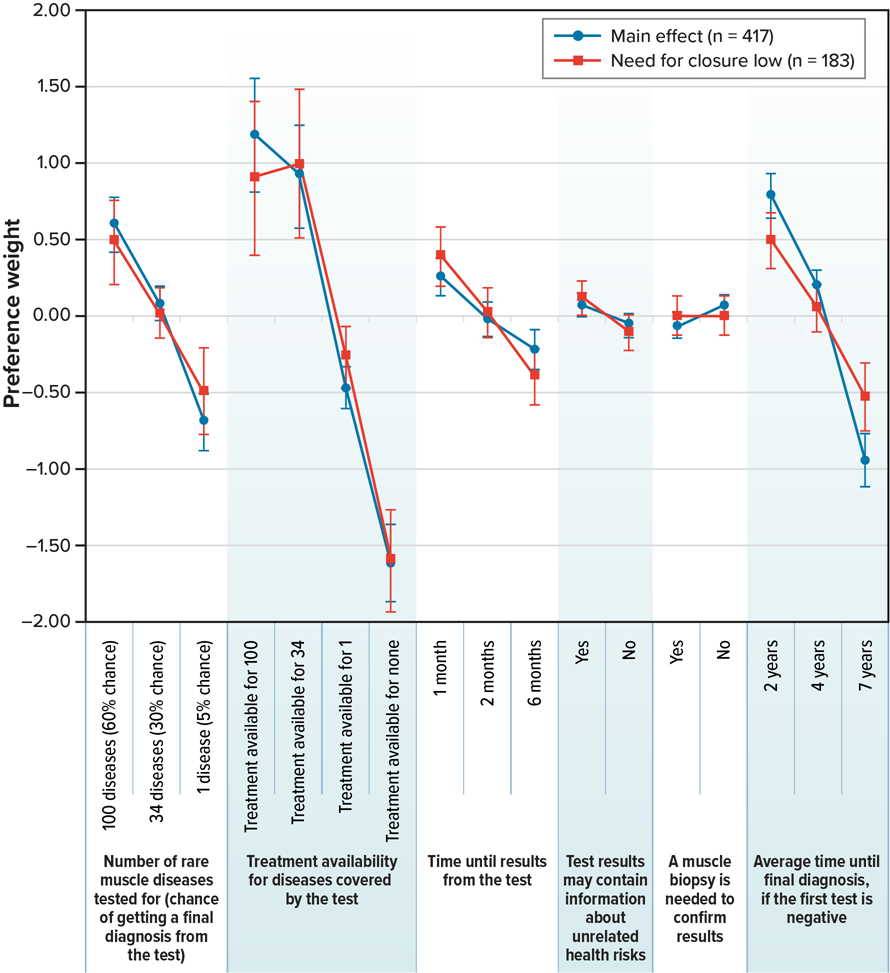


Note: The vertical bars surrounding each mean preference weight denote the 95% confidence interval of the point estimate.

Figure S-5. Conditional Relative Importance for the Subgroup Analysis Based on Whether Respondent Scored Low on the Need for Closure Ambiguity Scale or Not


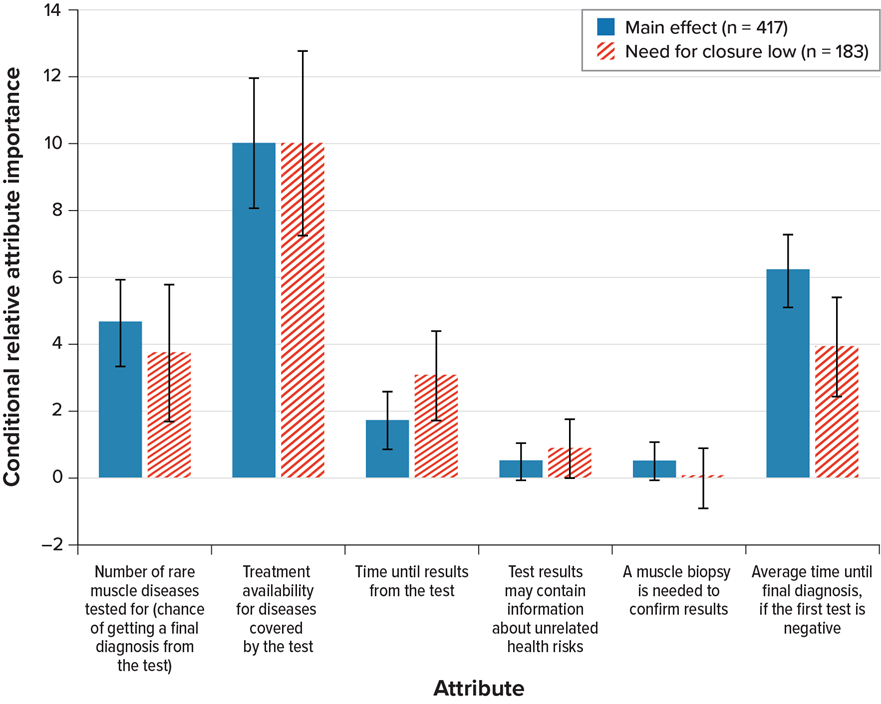


Note: The vertical bars surrounding each mean preference weight denote the 95% confidence interval of the point estimate.
